# Comparative Structural and Functional Analyses of the Fusiform, Oval, and Triradiate Morphotypes of *Phaeodactylum tricornutum* Pt3 Strain

**DOI:** 10.3389/fpls.2021.638181

**Published:** 2021-04-12

**Authors:** Ludovic Galas, Carole Burel, Damien Schapman, Marc Ropitaux, Sophie Bernard, Magalie Bénard, Muriel Bardor

**Affiliations:** ^1^Normandie University, UNIROUEN, INSERM, PRIMACEN, Rouen, France; ^2^Normandie University, UNIROUEN, Laboratoire Glycobiologie et Matrice Extracellulaire Végétale (Glyco-MEV) EA4358, Rouen, France; ^3^Institut Universitaire de France, Paris, France

**Keywords:** microalgae, diatom, morphotype, organelles, cytoskeleton, secretion, *Phaeodactylum tricornutum*, biofactory

## Abstract

The diatom *Phaeodactylum tricornutum* is a marine unicellular microalga that exists under three main morphotypes: oval, fusiform, and triradiate. Previous works have demonstrated that the oval morphotype of *P. tricornutum* Pt3 strain presents specific metabolic features. Here, we compared the cellular organization of the main morphotypes of the diatom *P. tricornutum* Pt3 strain through transmission electron and advanced light microscopies. The three morphotypes share similarities including spectral characteristics of the plastid, the location of the nucleus, the organization of mitochondria around the plastid as well as the existence of both a F-actin cortex, and an intracellular network of F-actin. In contrast, compared to fusiform and triradiate cells, oval cells spontaneously release proteins more rapidly. In addition, comparison of whole transcriptomes of oval versus fusiform or triradiate cells revealed numerous differential expression of positive and negative regulators belonging to the complex dynamic secretory machinery. This study highlights the specificities occurring within the oval morphotype underlying that the oval cells secrete proteins more rapidly.

## Introduction

During the last decades, knowledge regarding cell biology of eukaryotic model organisms like plants, yeast, animal cells have increased tremendously (Bezanilla, 2013; Martin, 2014; Mathur et al., 2017). In contrast, comprehension of cellular processes from the marine diatom *Phaeodactylum tricornutum* is still limited. *P. tricornutum* is an unicellular Stramenopile believed to have arisen *via* a serial endosymbiotic event in which a red microalga were engulfed by a heterotroph (Moustafa et al., 2009; Bowler et al., 2010; Prihoda et al., 2012), thus generating specific genomic features and metabolic pathways (Bowler et al., 2008; Keeling and Palmer, 2008). Indeed, a recent investigation of *P. tricornutum* genome revealed that a total of 3,170 genes (26%) are unique and specific to this organism (Rastogi et al., 2018). *P. tricornutum* also harbors a combination of genes and metabolic pathways that belongs either to the plant or animal kingdoms (C4 photosynthetic pathway and urea cycle, for example) (Butler et al., 2020). It is a photoautotrophic organism for which molecular tools as well as transformation methods have been developed (Apt et al., 1996; Zaslavskaia et al., 2000; Niu et al., 2012; Miyahara et al., 2013; Zhang and Hu, 2014; Karas et al., 2015). Indeed, genetic engineering such as gene silencing (De Riso et al., 2009), TALEN (Daboussi et al., 2014; Serif et al., 2017), and CRISPR/cas9 (Nymark et al., 2016; Mann et al., 2017; Serif et al., 2018; Slattery et al., 2018; Stukenberg et al., 2018) has been proven to be efficient in *P. tricornutum.* These tools should in the near future help in deciphering cellular processes and optimizing the commercial exploitation of *P. tricornutum*, which naturally synthesizes numerous compounds of interest like pigments and omega-3 (Cadoret et al., 2012; Sasso et al., 2012; Hamilton et al., 2015; Kuczynska et al., 2015; Butler et al., 2020). In addition, *P. tricornutum* has been recently used for biotechnological applications such as the production of biopharmaceuticals including monoclonal antibodies (mAbs) (León-Bañares et al., 2004; Mathieu-Rivet et al., 2014; Hempel and Maier, 2016; Dumontier et al., 2018; Rosales-Mendoza et al., 2020*).* For example, engineered *P. tricornutum* is able to produce recombinant human anti-Marburg virus mAbs (Hempel et al., 2017) and functionally glycosylated human anti-hepatitis B mAbs (Hempel et al., 2011; Hempel and Maier, 2012; Vanier et al., 2015, 2018). Even successful, industrial exploitation and commercialization are still rather limited due to the amount of mAbs produced. Thus, increasing the production yield is a prerequisite before any industrialization of algae-made mAbs. Such improvement requires a better comprehension of the cellular and metabolism processes as well as the secretion mechanism. In the future, metabolic engineering strategies could be envisioned and implemented as exemplified for the production of high-value plant triterpenoid production (D’Adamo et al., 2019) and increase of lipid accumulation (Zou et al., 2018).

*Phaeodactylum tricornutum* is atypical as it occurs naturally in at least three distinct morphotypes: oval, fusiform, and triradiate (Borowitzka and Volcani, 1978). A fourth cruciform morphotype has sometimes being described (Wilson, 1946; Lewin et al., 1958; He et al., 2014). However, the fusiform morphotype is the more frequent one. It can be morphologically transformed under specific culture conditions into the oval or triradiate one, morphotypes being able eventually to switch back to the fusiform morphotype (Borowitzka and Volcani, 1978). Such plasticity is likely to be due to the poorly silicified cell wall of the fusiform morphotype. In contrast, the oval morphotype contains organized silicified frustules (Vartanian et al., 2009). Indeed, *P. tricornutum* cell is encased by a rigid silica frustule comprised of two overlapping thecae (Epithecae and Hypothecae), each composed of a valve and accompanying the girdle band (GB) region. The raphe represents slits of the valves allowing the secretion of mucilage that is involved in cell motility and adhesion (Martin-Jézéquel and Tesson, 2013; Willis et al., 2013). Recently, a pairwise comparison of the transcriptomes of the three morphotypes from *P. tricornutum* Pt3 strain revealed that 1% of genes are differentially expressed between the fusiform and the triradiate mophotypes whereas more than 22 and 29% are differentially expressed when comparing the oval versus fusiform and the oval versus triradiate, respectively (Ovide et al., 2018). Among the differentially expressed genes in the oval morphotype, genes encoding proteins involved in stress responses like heat shock proteins and protein containing DER1-like domain are up-regulated (Ovide et al., 2018). Such results agreed with previous observations which conclude that the oval morphotype represent a resistance form to stresses and survive in unfavorable conditions such as hyposaline conditions, low temperature, low light (Gutenbrunner et al., 1994; De Martino et al., 2007, 2011; Bartual et al., 2008). In agreement, it has recently been reported that 68% of the differentially expressed genes compared to the other morphotypes were found to be up-regulated and involved in the biosynthesis of triglyceride, glucuronomannan and nucleotide pathways (Ovide et al., 2018). In addition, these RNA-Seq data suggest that several components of the secretory machinery are regulated in the oval morphotype suggesting specific protein release (Ovide et al., 2018). In this work, we compare the structural features, cellular organization and kinetics of protein release of the three main morphotypes of *P. tricornutum*, namely the fusiform, oval and triradiate.

## Materials and Methods

### Culture and Growth Conditions of *Phaeodactylum tricornutum*

Fusiform, oval, or triradiate morphotype enriched cultures of *P. tricornutum* Pt3 strain (CCAP 1052/1B; CCMP 2558) were generated as previously described (Ovide et al., 2018). *P. tricornutum* cells were grown at 19°C in 1 L flask on a 16/8 h light/dark cycle with light intensity of 68 μmol m^–2^ s^–1^. The nutritive medium was composed of 100% seawater (Instant Ocean) for the fusiform and triradiate morphotypes and of 10% seawater (Instant Ocean) for the oval morphotype. Sterilized by filtration through a 0.22 μm filter and autoclaved, seawater was then complemented with trace elements and 80 mg L^–1^ of sodium metasilicate (Na_2_SiO_3_) as previously reported (Baïet et al., 2011). The diatom cells were cultured under ambient air. CO_2_ from the air was the only available source of carbon.

### Ultrastructural Characterization of *P. tricornutum* Morphotypes Through Transmitted Electron Microscopy

High pressure freezing (HPF) was performed with the HPF-EM PACT I freezer from Leica Microsystems (Nanterre, France). Prior to freezing, cells were treated with 100 mM mannitol during 2 h at room temperature for cryopreservation. Pre-treated diatoms were then transferred into the cavity of a copper ring (diameter of 1.2 mm; depth of 100 μm). Using a horizontal loading station, the specimen carriers were tightened securely to the pod of specimen holder. After fixation on the loading device, specimen were frozen with a maximum cooling rate of 10,000°C s^–1^, an incoming pressure of 7.5 bars and a working pressure of 4.8 bars. Copper rings containing frozen samples were stored in liquid nitrogen until the freeze substitution procedure was initiated. After high-pressure freezing, samples were transferred to a freeze substitution automate (AFS, Leica Microsystems) pre-cooled to −140°C. As previously described (Ovide et al., 2018), samples were substituted in anhydrous acetone with 0.5% uranyl acetate at −90°C for 96 h. Using a gradient of +2°C h^–1^, the temperature was gradually raised from −90 to −15°C with two intermediate steps at −60 and −30°C. Finally, samples were rinsed twice with anhydrous ethanol.

Resin infiltration was processed at −15°C in a solution of ethanol/London Resin White (LRW) with successive ratios of 2:1 overday; 1:1 overnight and 1:2 overday followed by a final step in a pure LRW solution renewed twice during 48 h. The LRW was finally polymerized into the AFS apparatus at −15°C under ultra violet light during 48 h. Ultrathin sections (80 nm; ultracut UCT, Leica Microsystems) of diatoms were collected onto carbon-formvar-coated nickel grids. A classical staining using uranyl acetate and lead citrate was done before sections were observed in a Philips, FEI Tecnai 12 Biotwin transmission electron microscope operating at 80 kV, with ES500W Erlangshen CCD camera (Gatan).

### Structural Characterization of *P. tricornutum* Morphotypes Through Confocal Microscopy

For confocal microscopy, fluorescent labeling were performed on living or fixed *P. tricornutum* cells. After the different steps of labeling and rinsing, 5 μL of the diatom cell solution were deposited on a 35-mm glass bottom microwell dish (MatTek corporation) and covered with a small agar pad (Fisher, 0.3 g/20 mL) to stabilize microalgae during imaging. Acquisitions were performed at room temperature with an inverted Leica TCS SP5 confocal microscope (Leica Microsystems, Nanterre, France).

### Determination of Spectral Characteristics of *P. tricornutum* Cells Autofluorescence Through Confocal Microscopy

One-photon excitation (Ex) and emission (Em) spectra were measured at room temperature using Λλ acquisition mode on a TCS SP5 confocal microscope equipped with a supercontinuum laser source (NTK photonics, Cologne, Germany) and a resonant scanner (8,000 Hz). Using a 63× objective (1.4, oil immersion), autofluorescence emission from diatom cells was detected through a hybrid detector (Leica Microsystems, France). In this configuration, two-dimensional scanning with automatic variations of excitation (Λ, from 470 to 670 nm, 2 nm step) and emission (λ, from 490 to 800 nm, 10 nm band) was performed and led to a stack of 1,722 images (*n* = 20). Resulting Λλ representation, also called Lambda square fluorescence mapping, was obtained using the Excitation Emission Contour Plot of the Leica Application Suite Advanced Fluorescence software (Leica Microsystems, France). Therefore, each element of the mapping is defined by a corresponding couple of Ex/Em wavelengths. Excitation and emission spectra can therefore be obtained through Microsoft Excel. Consequently, autofluorescence emission of *P. tricornutum* cells was collected from 640 to 720 nm.

### Labeling of Living *P. tricornutum* Cells for Nucleic Acids, Mitochondria and Lipid Bodies

To avoid any spectral contamination between cells autofluorescence and green-emitted fluorescent probes for macromolecules and organelles, excitation and emission spectra were measured for Syto 21 and autofluorescence using Λλ acquisition mode as described above and spectral emission windows were determined for each fluorescent component.

For nucleic acids labeling, incubation with Syto 21 (Thermo Fisher Scientific) at a concentration of 10^–6^ M during 5 min, was performed in the respective nutritive medium for fusiform, triradiate, and oval morphotypes. For cell imaging, Syto 21 was excited at 488 nm and fluorescence was collected from 520 to 560 nm. As shown by the Λλ acquisition, activation of the 488 nm wavelength of the supercontinuum laser also induced simultaneous excitation of diatom cells autofluorescence that was detected between 640 and 720 nm. For mitochondria labeling, incubation with Mitotracker Green FM (Thermo Fisher Scientific) at a concentration of 10^–6^ M during 30 min was performed. For cell imaging, Mitotracker green was excited at 488 nm and fluorescence was collected from 500 and 550 nm. For lipid bodies labeling, incubation with BODIPY 505/515 (Thermo Fisher Scientific) at a concentration of 1 mg/mL during 10 min was performed. For cell imaging, BODIPY 505/515 was excited at 500 nm and fluorescence was collected from 510 and 550 nm. As shown by the Λλ acquisition, activation of the 500 nm wavelength of the supercontinuum laser also induced simultaneous excitation of cells autofluorescence that was detected between 640 and 720 nm.

### Labeling of Fixed *P. tricornutum* Cells for Actin

*Phaeodactylum tricornutum* Pt3 cells were fixed in 4% formaldehyde for 1 h and rinsed twice in phosphate buffered saline (PBS). After 3 min pre-incubation with 1% bovine serum albumin (BSA) and 0.1% Triton X-100 in PBS, cells were exposed to Alexa Fluor 488-conjugated phalloidin (165 nM, Invitrogen) for 30 min. For cell imaging, Alexa Fluor 488-conjugated phalloidin was excited at 488 nm and fluorescence was collected from 500 and 550 nm.All experiments have been performed at least from three different cell culture and representative images were chosen among at least 20 images to illustrate the different fluorescent labeling.

### Relative Quantification of Protein Release From *P. tricornutum* Pt3 Morphotypes

*Phaeodactylum tricornutum* cells (2.10^5^ cells mL^–1^) were used to inoculate eight flasks for each morphotype, in 100% seawater medium (Instant Ocean) for the triradiate and fusiform morphotypes and 10% for the oval one, respectively. The medium and the culture conditions were as described in the section “Culture and Growth Conditions of *Phaeodactylum tricornutum*.” Each day and for each morphotype, the number of cells was counted in order to establish a growth curve. For each day of culture, culture medium from one flask were recovered by centrifugation at 4,500*g*. Cell pellets were discarded and supernatants containing the culture medium were harvested, dialyzed and lyophilized. The samples were then resuspended in the same volume of milliQ water. To profile the protein release from the different morphotypes, proteins contained in the culture media were separated on a Sodium Dodecyl Sulfate-polyacrylamide gel electrophoresis (SDS-PAGE). For each morphotype and each day, the volume of secreted medium equivalent to 7.6 × 10^6^ cells were loaded after denaturation using a Laemmli buffer on a SDS-PAGE, ran in a Bis-Tris gel 4-12%. Secreted proteins are finally revealed by silver staining. A 8-bit tiff image of the gel obtained using the Fusion FX6 acquisition system with eVo-6 camera (Vilber). ImageJ (Abràmoff et al., 2004; Rasband, 1997–2018), was used to perform relative quantification of silver-stained proteins. Tiff image was first inverted to finely localize specific staining within an appropriate region of interest (ROI). Subtracted from background noise, the sum of pixel intensity for each ROI was calculated and considered as the indicator of total protein content for each day of culture. All values were normalized by the maximum value detected in the gel i.e., day1 for oval cells and expressed as a kinetic of protein release over days.

### Image Analysis

Deconvolution of raw data from confocal imaging was obtained through image processing with Huygens professional 4.5.1 sofware (SVI). ImageJ was used to adjust image brightness and contrast and to perform z projections of 3D images (xyz).

### Transcriptome Analysis

The transcriptomic full dataset from Ovide et al., 2018 comparing the oval versus fusiform and the oval versus triradiate cells were combined and were manually reinvestigated in order to identify and select mRNA encoding for fucoxanthin chlorophyll a/c, proteins involved in actin and tubulin network, for signal peptidases and signal recognition particle proteins and finally proteins involved in vesicular trafficking. Supplementary Tables 1, 2 were build based on these analyses.

## Results and Discussion

The Pt3 strain was adapted to generate enriched cultures in each specific morphotype as previously described in Ovide et al. (2018). Morphotypes were studied and compared with respect to organelles and kinetics of protein secretion.

### Ultrastructural Characterization of Pt3 Cells Through Transmission Electron Microscopy

**FIGURE 1:**
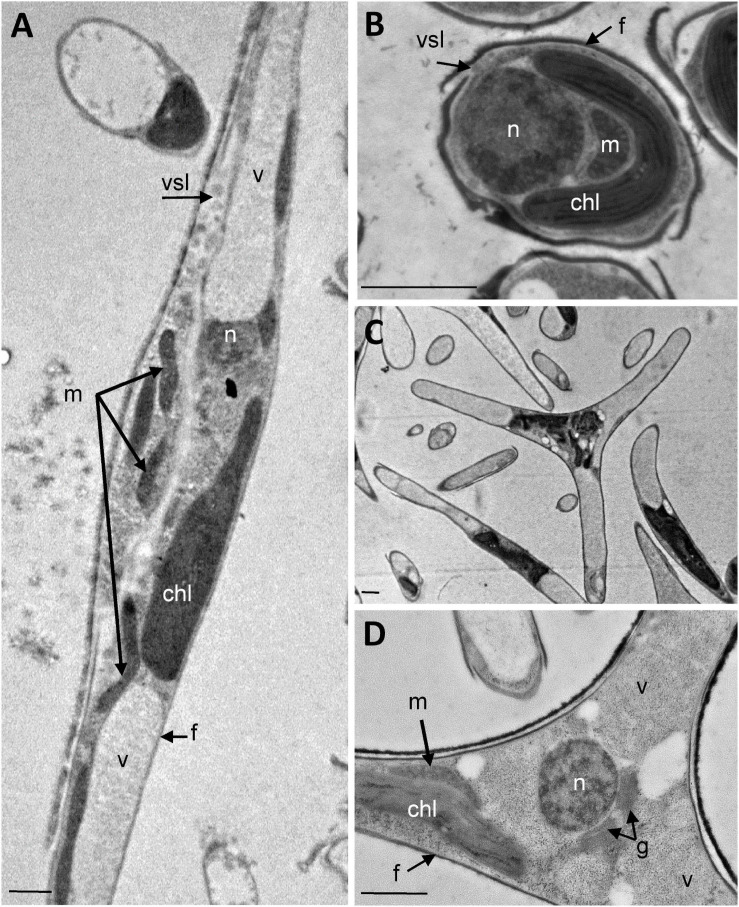
Ultrastructural characterization of *P. tricornutum* Pt3 morphotypes. Transmission Electron micrographs of *P*. *tricornutum* Pt3 cell morphotypes. Overview of the cells embedded in LRW resin with 0.5% uranyl acetate in a methanol/Reynold’s lead citrate solution. **(A)** Fusiform morphotype; **(B)** Oval morphotype, and **(C)** Triradiate morphotype. **(D)** Enlarge view showing cellular distribution organelles in triradiate morphotype. chl: plastid; f: frustule; g: Golgi apparatus; m: mitochondria; n: nucleus; vsl: vesicle; v: vacuole;. Bars, 1 μm.

The analysis of the ultrastructure of the three morphotypes was performed by transmission electron microscopy (TEM). Electron micrographs of *P. tricornutum* fusiform, oval and triradiate morphotypes are shown in Figure 1. As expected, the sections reveal cells surrounded by the frustule which is poorly silicified in the fusiform and triradiate cells (Figures 1A,C,D), compared to the oval cells (Figure 1B; Borowitzka and Volcani, 1978; Francius et al., 2008; Tanaka et al., 2015). Overall, similar organelles were found in the three morphotypes (Figure 1) including nucleus (n), plastid (chl), mitochondria (m), vacuoles (v), vesicles (vsl). Vacuoles are larger in the fusiform and triradiate cells (Figures 1A,D). They occupy the distal arms of the cells. A single and large plastid is present and localized nearby the nucleus in the three morphotypes. When observed, Golgi apparatus can be found closed to the nucleus (Figure 1D). Mitochondria are elongated and generally reach both extremities of the cells, especially in the fusiform and triradiate cells (Figures 1A,D). Such observations correlate with previous description (Martin-Jézéquel and Tesson, 2013) and validate the integrity of *P. tricornutum* cells in the culture conditions used for this work. Then, the three morphotypes of *P. tricornutum* were further characterized with advanced light microscopy by taking advantages of cellular autofluorescence and labeling of living cells using specific fluorescent organelles probes.

### Spectral Characterization of Plastid Autofluorescence in Pt3 Cells

**FIGURE 2:**
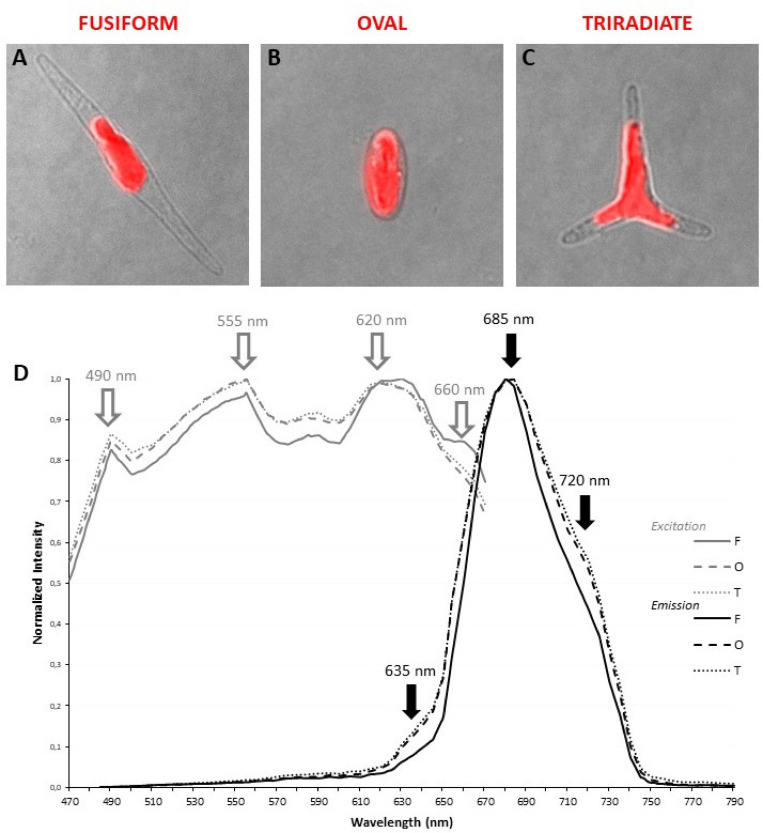
Localization and spectral characterization of plastid autofluorescence in living *P. tricornutum* Pt3 morphotypes through confocal microscopy. Merged images (transmitted light and confocal microscopy) illustrating the shape and the localization of autofluorescent plastid in oval **(A)**, fusiform **(B)**, and triradiate **(C)** morphotype. **(D)** Excitation/emission spectra of plastid autofluorescence were obtained with a supercontinuum laser source through Λλ-scan microscopy (*n* = 25). Excitation spectra are represented by gray lines. Emission spectra are represented by black lines. Dashed lines: oval cells; solid lines, fusiform cells; dotted lines: triradiate cells.

Living *P. tricornutum* cells contain a single and large autofluorescent plastid whose shape is related to the cell organization of each morphotype. In fusiform cells, the organelle is central and presents an elongated aspect (Figure 2A) while in triradiate cells, the plastid is located in a central position and is extended in the initial part of the three distal arms (Figure 2C). In oval cells, the ovoid plastid occupies a large portion of the cell volume (Figure 2B). Thanks to the supercontinuum laser and the Lambda Square mode for signal detection, simultaneous 1P excitation (Λ) and emission (λ) spectra were obtained for plastid autofluorescence in living cells at room temperature. As described in the Materials and Methods section, the two-dimensional scanning with automatic variations of excitation (Λ, from 470 to 670 nm, 2 nm step) and emission (λ, from 490 to 800 nm, 10 nm band) is particularly appropriate to define robust fluorescence imaging settings for multi-labeling experiments. In contrast, this approach will not allow the discrimination of individual pigments i.e., within excitation profile that requires a single emission wavelength at very low temperature as proposed by Lamote et al. (2003). In the current study, three major peaks of excitation around 490, 555, and 620 nm are observed for all three morphotypes (Figure 2D). It is assumed that carotenoids are excited at 490 nm, fucoxanthin at 555 nm while chlorophyll *c* presents a major peak of excitation around 620 nm (Lamote et al., 2003, Veith and Bühel, 2007). In addition, a slight excitation of chlorophyll *a/c* with a 490-nm laser cannot completely be ruled out. The fusiform morphotype presents an additional shoulder in its excitation profile above 660 nm. For emission, a major 130 nm-band (full width at half maximum) between 660 and 730 nm with a maximum at 685 nm could be detected and attributed to the light energy collecting complex of photosystem II (PSII) as previously proposed for *Fucus serratus* 8 h-old embryos when excited at 440 nm (Lamote et al., 2003). These results are also consistent with previous data demonstrated that some fucoxanthin chlorophyll *a*/*c* proteins of *P. tricornutum* cells as components of PSII (Levitan et al., 2019) emitted at 683 nm when excited by single laser lines at 473 or 532 nm (Premvardhan et al., 2013). In addition to the major emission peak, two additional shoulders around 630 and 720 nm, respectively, are observed for oval and triradiate morphotypes. The shoulder at 630 nm might reflect that chlorophyll *c* is not integrated within the PSII complex whereas the one at 720 mn suggests that the ratio of PSI/PSII and/or content of lhcf15 could be higher in the oval and triradiate morphotype compare to the fusiform one (Lamote et al., 2003; Herbstová et al., 2017). Whether the latest is related to enhanced energy transfer mechanisms, to an increase in PSI complex or in lhcf15 deserve further investigations. The emission at 720 nm might also be the result of stress conditions (Premvardhan et al., 2013), which is coherent with the fact that oval cells are preponderant under unfavorable growth conditions (De Martino et al., 2007, 2011). *P. tricornutum* genome encodes 42 predicted light-harvesting complex (LHC) or fucoxanthin chlorophyll *a*/*c* proteins (Depauw et al., 2012; Nymark et al., 2013; Levitan et al., 2019).^1^ Among them, transcriptomic analysis revealed that some genes like Phatr3_J32294 (lhcr8; UniProt: B7FQS0), Phatr3_J10243 (lhcr9; UniProt: B5Y4K0), Phatr3_J30643 and Phatr3_J29266 (lhcf6; UniProt: B7G5S7), Phatr3_J30031 (lhcf9; UniProt: B7G955), Phatr3_J18049 (lhcf1; UniProt: B7FRW5), and Phatr3_J25172 (lhcf2; UniProt: B7FRW4) are differentially overexpressed in the oval morphotype when compared to the fusiform and triradiate ones (Ovide et al., 2018). Expression of genes like Phatr3_J46529 encoding extrinsic protein in Photosytem II (UniProt: B7G1J1), Phatr3_J11006, and Phatr3_J42519 encoding lhcr1 (UniProt: B7FUM6) and fucoxanthin chlorophyll binding protein related (UniProt: B7FRK1), respectively, are also up-regulated in the oval cells compared to the fusiform and triradiate morphotypes. Accumulation of fucoxanthin had already been described in *P. tricornutum* under low light intensities and depending of the culture conditions (Gómez-Loredo et al., 2016; McClure et al., 2018). Moreover, in diatoms, chlorophyll *a* fluorescence could change as a result of external stimulants or growth phase (Kuczynska et al., 2015) and autofluorescence spectral characteristics can therefore be considered as a “health indicator” during biotechnological applications. Fluorescence Life-time Imaging Microscopy (FLIM) might be further considered to discriminate autofluorescence components and variations as previously proposed by Kuczynska et al. (2015).

### Localization of Nucleic Acids Materials in Pt3 Cells

**FIGURE 3:**
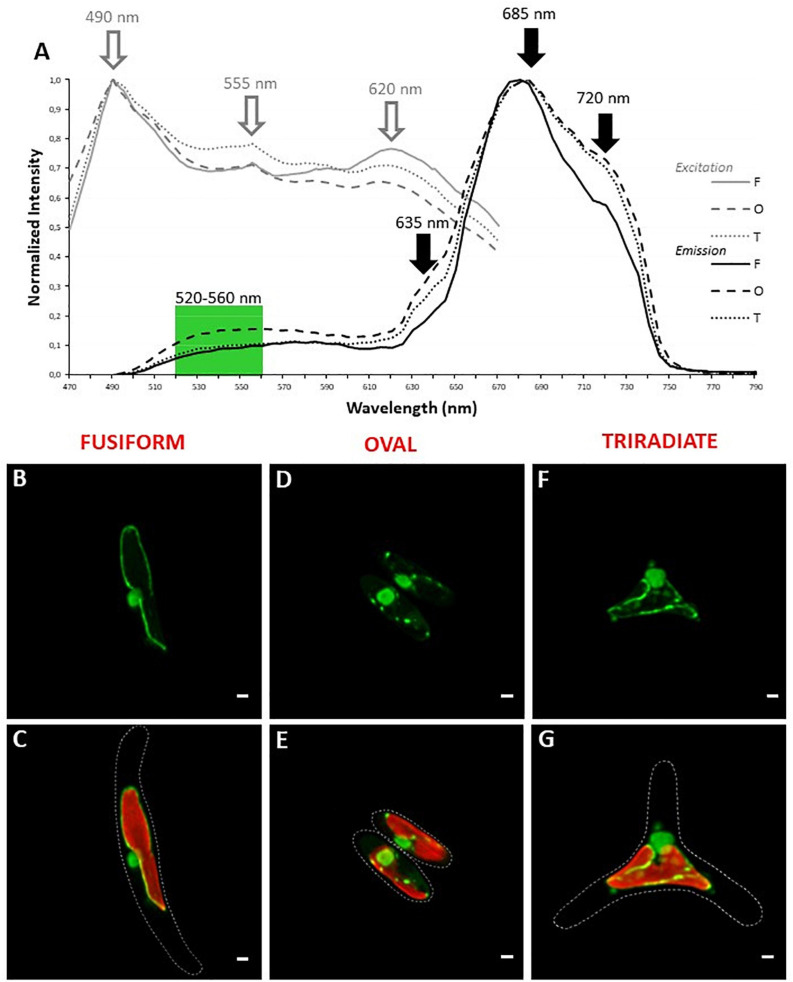
Localization of nucleic acids in living *P. tricornum* Pt3 morphotypes. **(A)** Spectral characterization (Λλ-scan microscopy) for single excitation and double emission settings in Syto 21-labeled and autofluorescent choloroplast-containing cells (*n* = 20). Excitation spectra are represented by gray lines. Emission spectra are represented by black lines. Dashed lines: oval cells; solid lines, fusiform cells; dotted lines: triradiate cells. **(B,D,F)** Localization of DNA/RNA in Pt3 morphotypes after staining with Syto 21. **(C,E,G)** Combination of Syto 21 images with plastid autofluorescence. Bars, 1 μm.

Since *P. tricornutum* plastid autofluorescence exhibits complex spectra for excitation and emission with multiple peaks or shoulders. Simultaneous detection of autofluorescence and other labeling were performed with green light emitting markers only. In addition, similar simultaneous 1P excitation (Λ) and emission (λ) approach was replicated, at least with a DNA/RNA green fluorescent marker named Syto 21 (excitation/emission 494/517 nm), to determine robust spectral configuration for simultaneous fluorescent detection. Similar peaks of excitation at 490, 550, and 620 nm were obtained. Interestingly, the 490 nm-peak, also described as an excitation wavelength for Syto 21, becomes predominant (Figure 3A). As expected, autofluorescence emission peaks at 685 and 720 nm were detected but an additional large band of emission from 510 to 600 nm was observed for Syto 21 (Figure 3A). Consequently, single excitation at 490 nm and sequential detection between 520–560 nm and 640–720 nm were used for Syto 21 and autofluorescence, respectively. In these conditions, a central rounded nucleus is observed next to the plastid in all three Pt3 morphotypes (Figures 3B,D,F) as previously described for other strains (Borowitzka and Volcani, 1978; Siaut et al., 2007; Tanaka et al., 2015; Flori et al., 2017). In addition to nuclear staining, Syto 21-positive materials were also detected around the plastid. In particular, punctiform and sparse Syto 21-positive elements were distributed close to the plastid in oval cells (Figure 3C). In fusiform cell, Syto 21-labeling finely delimits the plastid (Figure 3E) while staining in triradiate cells was a mix of oval and fusiform ones with both punctiform elements and plastid outlining (Figure 3G). Since Syto 21 recognizes both DNA and RNA, extra-nucleus labeling may represent either endoplasmic reticulum (ER) and/or mitochondrial DNA. This is in agreement with the fact that the nuclear envelop has been described to be part of the ER surrounding the plastid (Borowitzka and Volcani, 1978). Moreover, when ER specific proteins like SEC61 subunit or the hDER 1, a central component of the ERAD machinery, were expressed in *P. tricornutum* as eGFP fusion proteins, their localization highlight the ER, the nuclear envelope as well as the outermost membrane of the complex plastid (Liu et al., 2016).

### Localization of Mitochondria in Pt3 Cells

**FIGURE 4:**
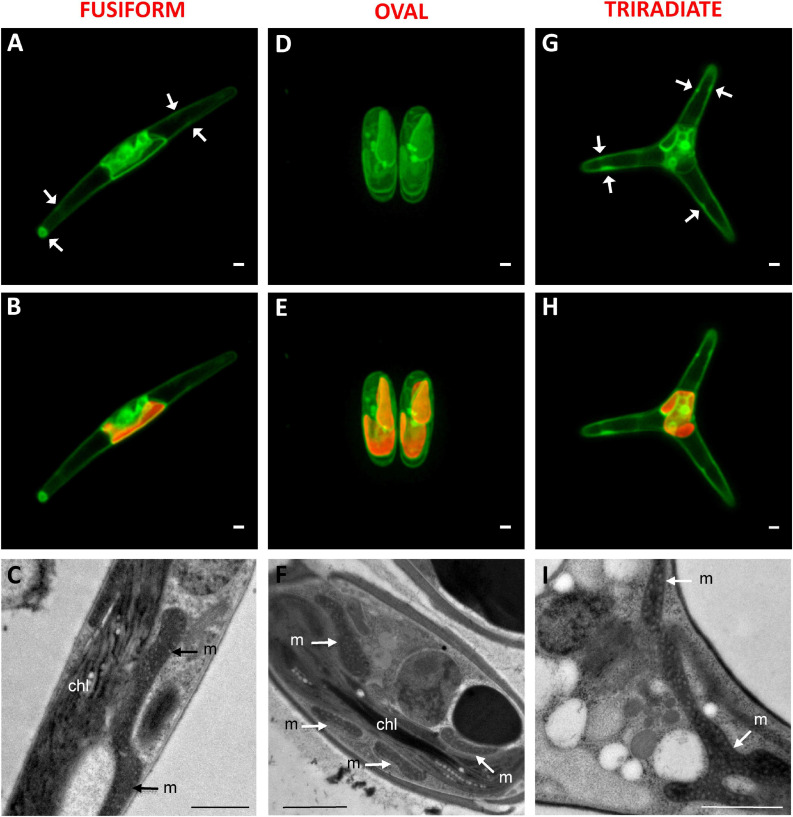
Localization of mitochondria in living *P. tricornutum* Pt3 morphotypes. **(A,D,G)** Localization of mitochondria in Pt3 morphotypes in living cells after staining with Mitotracker Green in the fusiform morphotype **(A)**, oval morphotype **(D)**, and triradiate morphotype **(G)**. White arrows indicate peripheral spots of Mitotracker Green staining. **(B,E,H)** Combination of Mitotracker Green images with plastid autofluorescence in the three Pt3 morphotypes. Enlarged views of tubular mitochondria on Transmission Electron micrograph of each morphotype **(C,F,I)**. chl: plastid; m: mitochondria. Bars, 1 μm.

Mitochondria distribution in living *P. tricornutum* cells was studied with the Mitotracker Green probe as previously used for labeling fusiform cells in Liu et al. (2016). In all morphotypes, a moderate to intense Mitotracker Green positive signal delimits the outline of the plastid (Figure 4A,B,D,E,G,H). This is in agreement with transmission electron micrographs where tubular mitochondria can be observed close to the plastid in the oval (Figure 4C), fusiform (Figure 4F), and triradiate cells (Figure 4I). Similarly, transgenic *P. tricornutum* Pt1 expressing mitochondrial targeting glutamine synthetase III fusion protein displayed eYFP signal that surrounded the plastid (Siaut et al., 2007). Expression of a mitochondrion marker like a subunit of the glycine decarboxylase complex as an eGFP-fusion protein resulted in a fluorescence pattern near the complex plastid in the fusiform cells (Liu et al., 2016). Moreover, in *P. tricornutum* Pt1 fusiform cells, a continuous network of mitochondria sitting on the plastid is also clearly described through focused ion beam-scanning electron microscopy (Bailleul et al., 2015; Flori et al., 2017, Uwizeye et al., 2020). Such physical contacts between the two organelles may possibly facilitate exchange of energy. In this study, Mitotracker Green-staining was also widely detected in the cytoplasm of the Pt3 cells generally close to the plastid but also within distal arms of fusiform and triradiate morphotypes as peripheral spots (white arrows) (Figures 4A,G). Similar elongated branched mitochondrion is also described in fusiform Pt1 cells during interphase (Tanaka et al., 2015) or in tomograms of Pt1 (Uwizeye et al., 2020). Intriguingly, incubation with Mitotracker Green also induced a fluorescence signal along the plasma membrane of oval, fusiform and triradiate cells, suggesting numerous elongated mitochondria at the cell periphery as confirmed by the TEM observation (Figures 4C,F,I). Mitotracker Green was chosen in this study for spectral considerations but differential distribution of mitochondria may be noted with different fluorescent Mitotrackers (Galas et al., 2018). In particular, fluorescence of the Mitotracker Orange and Red probes is dependent of mitochondrial potential while Mitotracker Green is not. Therefore, activity of mitochondria observed close to the plasma membrane as shown in electron micrographs (Figures 1, 4), might be different compared to others observed at the vicinity of plastids. Alternatively, abnormal adsorption of Mitotracker Green on the frustule cannot be completely ruled out since no labeling for nucleic acids belonging to the mitochondrial genome was observed in this area.

### Localization of Lipid Bodies in Pt3 Cells

**FIGURE 5:**
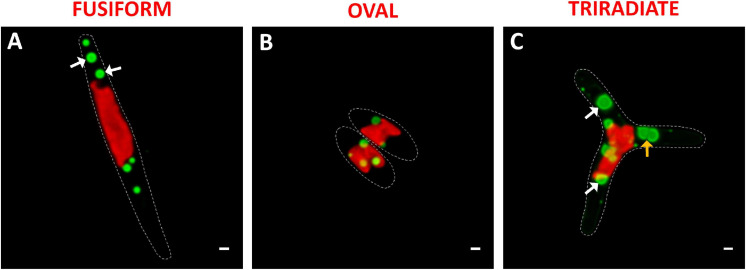
Localization of lipid droplets in living *P. tricornutum* Pt3 morphotypes. **(A–C)** Localization of lipid bodies in Pt3 morphotypes after staining with Bodipy 505/515. Combination of Bodipy 505/515 images with plastid autofluorescence. White arrows indicate lipid bodies with very clear outer ring. Orange arrow illustrate fusion between two lipid bodies. Bars, 1 μm.

*Phaeodactylum tricornutum* microalgae synthesize and store neutral lipids mainly triglycerides in lipid bodies also called lipid droplets (Wong and Franz, 2013; Lupette et al., 2019). The distribution of lipid droplets was studied in living *P. tricornutum* Pt3 cells using Bodipy 505/515, which has a small fluorescence Stokes shift and high fluorescence quantum yield for lipids. At this stage of Pt3 culture (day 8), lipid bodies are spherical (Figure 5). This contrasts to previous observation of *P. tricornutum* aging culture in which lipid bodies appear as single or double large ovoid lipid droplets (Wong and Franz, 2013). This depends on growth conditions and carbon availability. In this work, small lipid droplets were generally distributed close to the plastid in living oval cells (Figure 5B). In contrast, bigger and more numerous lipid bodies were detected in the fusiform and triradiate cells (Figures 5C,D). This agrees with a previous report that described lipid droplets in contact with chloroplast (Lupette et al., 2019). The lipid bodies were observed in the distal arms of living fusiform cells as middle size lipid organelles (Figure 5A), whereas living triradiate cells contained lipid bodies around the plastid and in distal arms with a large size scale from punctiform to large droplets (Figure 5C). In Pt3 cells, lipid bodies are delimited by a ring (white arrows). Previous studies indicate that in *P. tricornutum*, droplets tend to fuse leading to a restricted number of large lipid bodies while in *Tetraselmis suecia* new lipid bodies are synthetized (Wong and Franz, 2013). A possible merge between two lipid droplets is indicated by an orange arrow in Figure 5C. From this work, it appears that the Pt3 triradiate cells seems to possess bigger neutral lipids droplets. Interestingly, an additional cruciform morphotype of *P. tricornutum* resulting from triradiate cells transformation with low temperature culture conditions presented a unique fatty acids characteristics suitable for biodiesel production (He et al., 2014). In 2020, Song et al. (2020) observed more and larger lipid bodies in Pt1 and Pt4 fusiform cells over time compared to oval cells. This implies higher neutral lipid accumulation in the fusiform cells from these *P. tricornutum* strains (Song et al., 2020).

### Comparison of the Secretory Potential of Pt3 Fusiform, Oval and Triradiate Morphotypes

**FIGURE 6:**
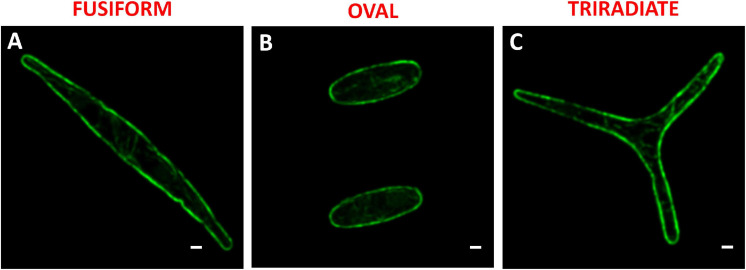
Localization of actin in fixed *P. tricornutum* Pt3 morphotypes. **(A–C)** Localization of F-actin in Pt3 morphotypes after staining with Alexa 488-coupled phalloidin. Bars, 1 μm.

In diatoms, the molecular mechanisms underlying the release of vesicles including silica deposition vesicles for generation of the silicified frustule (Siaut et al., 2007) or antibody/protein-containing vesicles (Hempel et al., 2011, 2017; Vanier et al., 2015, 2018), are not yet understood and rarely investigated (Erdene-Ochir et al., 2019). In particular, the involvement of cytoskeleton elements in the context of the secretory pathway is poorly described. In this study, the localization of F-actin was determined in fixed permeabilized *P. tricornutum* cells with Alexa 488-Phalloidin. In all three morphotypes, an intense Alexa 488-phalloidin positive signal delimits the outline of the cell indicating the existence of an actin cortex under the plasma membrane (Figure 6). Cortical labeling for Syntaxin-A and Sec4 was also observed in Pt1 fusiform cells, suggesting the existence of regulated mechanisms of vesicle fusion and secretion in *P. tricornutum* (Siaut et al., 2007; De Martino et al., 2011). An actin network with a faint fluorescent signal was detected in the cytoplasm of fusiform, oval and triradiate cells (Figure 6) suggesting a role of actin in vesicular trafficking together with small GTPase such as SEC4 (Siaut et al., 2007; De Martino et al., 2011). In addition, re-analysis of whole transcriptomic dataset from Ovide et al., 2018 reveals that genes encoding components of the actin network like Phatr3_J44183 encoding the actin cortical patch component lsb4 (UniProt: B5Y5L8) is down-regulated (−1.5 fold) whereas Phatr3_J9601 encoding F-actin capping protein subunit β (UniProt: B7FPL9) and Phatr3_J35252 encoding F-actin capping protein (UniProt: B7FXZ8) are up-regulated in the oval morphotype compared to the fusiform and triradiate ones (+1.1 and +1.7 fold, respectively). Other genes encoding for molecular actors associated to actin like Phatr3_J20837 encoding the actin-related protein 4 (no UniProt number available), Phatr3_J48922 encoding condensin complex subunit 3 (UniProt: B7G8V9), genes encoding myosin proteins (Phatr3_EG02335, UniProt: C6JVY2; Phatr3_J52058, UniProt: C6JVY4; Phatr3_J432, UniProt: C6JVY6) are also down-regulated in the oval cells. In contrast, Phatr3_J45476 encoding villin-3-like isoform x1 (UniProt: B7FXU1), Phatr3_J53980 encoding the gelsolin-like protein 2-like (UniProt: B7FPI9), Phatr3_EG02110, UniProt: C6JVY3; Phatr3_EG02422, UniProt: C6JVY5; Phatr3_EG0237 (UniProt: C6JVY7), and Phatr3_J25867 (UniProt: C6JVY8) encoding proteins from the myosin complex and gene encoding the cofilin tropomyosin-type actin-binding protein (Phatr3_EG00210) are up-regulated in the oval cells (Ovide et al., 2018). More experimental work will be needed in the future to decipher the secretion mechanism in *P. tricornutum*.

Despite extensive efforts through immunocytochemistry or silicon rhodamine probe strategies (Galas et al., 2018), we never manage to observe microtubules in *P. tricornutum* as similarly mentioned by Tanaka et al. (2015). This contrast with previous report claiming the observation of microtubules near the nucleus during division of *P. tricornutum* (Borowitzka and Volcani, 1978), *Coscinodiscus granii* and *Entomoneis alata* (Tesson and Hildebrand, 2010). When comparing whole transcriptomes of oval versus fusiform or triradiate cells, only Phatr3_J44333 encoding the gamma-tubulin complex component 3, Phatr3_J17048 encoding the tubulin-specific chaperone a (Uniprot: B7GEH7), and Phatr3_J37751 encoding the tubulin-tyrosine ligase (Uniprot: B7G3N2) have been identified to be slightly overexpressed (between 1.7- and 2.9-fold, respectively) in the oval cells (Ovide et al., 2018). In contrast, many proteins from the kinesin complex and other proteins, which are known to move along or to be associated with the microtubules (Supplementary Table 1) are differentially expressed in the oval cells (Ovide et al., 2018). Further investigations need to be performed in order to evaluate whether tubulin components participate to the vesicle trafficking and release in *P. tricornutum*.

**FIGURE 7:**
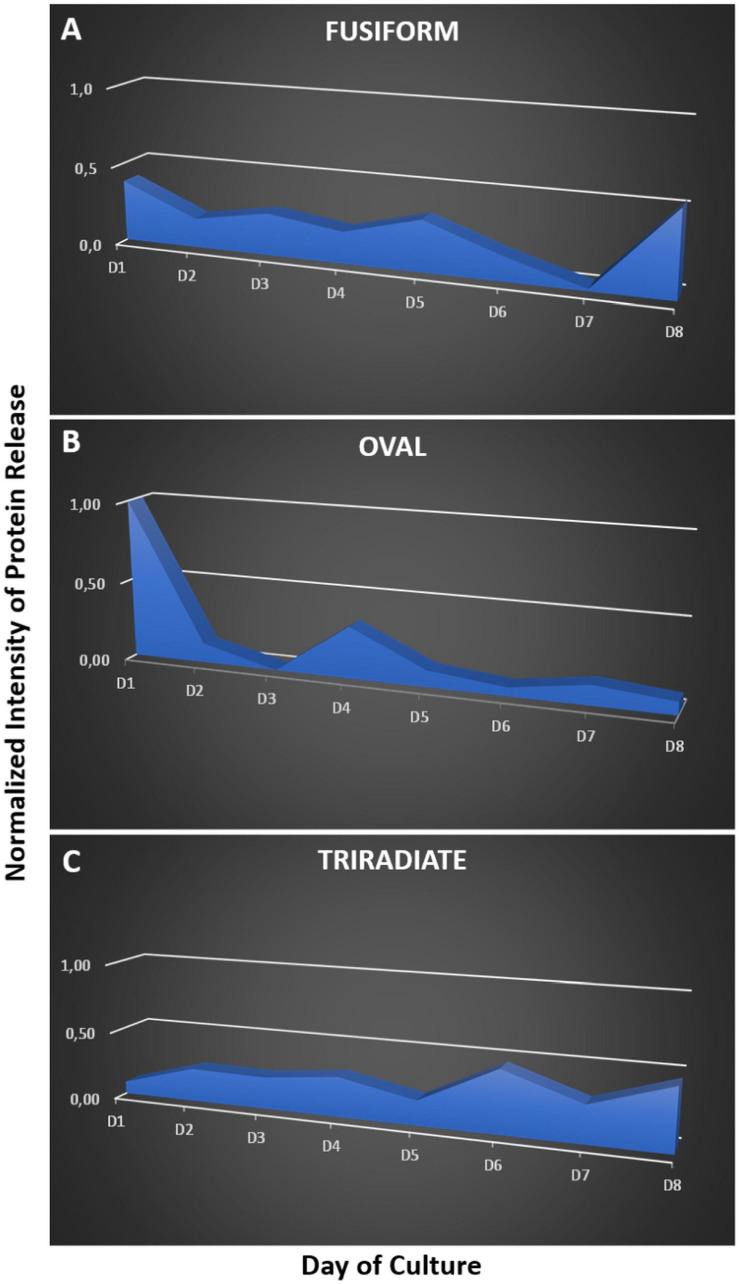
Kinetics of protein release by the three morphotypes of *P. tricornutum* Pt3 strain over a 8 days period of culture. The proteins present in the culture medium of fusiform **(A)**, oval **(B)**, and triradiate **(C)** were separated by SDS-PAGE. Proteins in the gel were labeled with silver staining. Relative quantification of stained proteins were performed with ImageJ.

So far, *P. tricornutum* cells are known to secrete adhesive mucilage also called exopolymeric substances or EPS. EPS secretion occurs from the GB region in the oval cells. EPS are excreted by the three morphotypes of *P. tricornutum* but significant variations have been observed in the EPS composition, especially between the fusiform and oval cells (Willis et al., 2013). Moreover, secretion of extracellular components like laminarine, elastin, fibronectin, mucin, tenascin have been suggested (Scala et al., 2002; Sapriel et al., 2009). In addition, the capacity of *P. tricornutum* to secrete proteins was highlighted toward proteomic analyses of the culture media. Such analyses allowed the identification of the most abundant proteins, 36 proteins in Buhmann et al. (2016) and 468 proteins in Erdene-Ochir et al. (2019), respectively. Among the secreted proteins in the culture medium of *P. tricornutum* cells, the “highly abundant secreted protein 1” (HASP1; Uniprot: B7G4A0) also called phosphate alkaline was confirmed by LC-MS/MS (Buhmann et al., 2016; Erdene-Ochir et al., 2019). The HASP1 signal peptide drives the entry of protein into the secretory pathway (Erdene-Ochir et al., 2019). Moreover, when the recombinant mAb directed against the hepatitis B was expressed in *P. tricornutum* cells, the human signal peptide from both the heavy and light chains were cleaved off in the diatom cells suggesting that they used a signal peptide peptidase mechanism that is similar to the one occurring in other eukaryotes (Vanier et al., 2015). All the past studies were performed on the fusiform cells independently of the morphotype of the *P. tricornutum* cells. In this work, we checked on previous transcriptomic dataset regarding the comparative mRNA expression between oval and fusiform and triradiate morphotypes, respectively by looking at the putative signal peptidases involved in the removal of the signal peptides (Ovide et al., 2018). This search allows the identification of Phatr3_J18533, Phatr3_J51280, Phatr3_J15399 and Phatr3_J13921 genes encoding respectively four signal peptidases (Uniprot: B5Y4T0; B7GDX7, B7G8T6, B7G343) that are overexpressed between 2.6- to 4.2-fold when comparing the oval versus fusiform and triradiate cells, thus suggesting that the oval cells present higher secretion capacity. Phatr3_J44439 gene encoding the signal recognition particle 19kDa protein (Uniprot: B7FU41) is also overexpressed 3.5 fold in the oval cells as compared to the fusiform and triradiate cells. In contrast, signal recognition particle proteins like the one encoded by Phatr3_EG02041 gene, the SRP54 encoded by Phatr3_J13417 gene (Uniprot: B5Y444) and the signal recognition particle 72 kDa protein encoded by Phatr3_J48508 gene (Uniprot: B7G7I1) are slightly down-regulated (less than 2 fold; Ovide et al., 2018). In addition, Phatr3_J47612 gene encoding the HASP1 protein (Uniprot: B7G4A0) is up regulated more than 4 fold in the oval cells compared to the fusiform and triradiate cells. In this context, in order to compare the kinetics of protein secretion of the three morphotypes, the spontaneous protein release in the culture medium of each morphotype was followed over a culture period of 8 days using SDS-PAGE (Figure 7 and Supplementary Figure 1). Such analysis highlights different kinetics of protein release between Pt3 cells. Indeed, the fusiform cells secreted proteins rather constantly (Figure 7A). In contrast, within the first 4 days, oval cells released more than 80% of their proteins while triradiate cells secreted only 38% (Figures 7B,C). In addition, triradiate cells increased regularly their secretory activity over the 8-days period (Figure 7C). Taken together, these data suggest differences in secretory kinetics between Pt3 cells. Oval cells are able to release rapidly a higher amount of proteins, fusiform cells present a constant secretory activity at a mid-level while triradiate cells release progressively proteins over time from low-level to mid-level. Such results are complementary to recent findings published by Song et al. (2020) that quantified higher protein content in oval cell cultures than in fusiform cell cultures for both Pt1 and Pt4 strains of *P. tricornutum*. In addition, as previously reported in Ovide et al. (2018), RNA-Seq transcriptomic analysis performed on the three morphotypes of *P. tricornutum* Pt3 strain highlighted, in the oval morphotype, overexpression of genes encoding proteins involved in vesicular transports like the SAR1, a GTPase found in COP II vesicles; BET1 a Golgi vesicular transport from the ER to the Golgi complex; the SNARE SEC22 and the syntaxin 6, which displays important role in protein trafficking between the *trans-*Golgi network and the endosomal system. In this study, other genes encoding proteins involved in vesicular trafficking are observed to be up and down-regulated in the oval morphotype (Supplementary Table 2). This includes genes encoding Clathrin-heavy chain (Phatr3_EG01984, UniProt: B7G4Y3), COP I (Phatr3_J49956, UniProt: B7GCF6) and COP II (Phatr3_J49955, UniProt: B7GCF; Phatr3_J47710, UniProt: B7G4M2) that are down-regulated. Several genes encoding ARF and RAB-related proteins have been identified to be down- or up-regulated suggesting a fine-tune regulation of the secretion in the oval cells (Supplementary Table 2). Interestingly, genes encoding coatomers like Phatr3_J19093 gene that encodes the coatomer subunit epsilon (Uniprot: B7FUJ7) and Phatr3_J7018 gene that encode the coatomer subunit zeta-1 (Uniprot: B7G7H3) are up-regulated in the oval cells. These coatomers might be involved in the retrograde vesicle-mediated transport from Golgi apparatus to ER. Such results might be confirmed experimentally in future studies.

**FIGURE 8:**
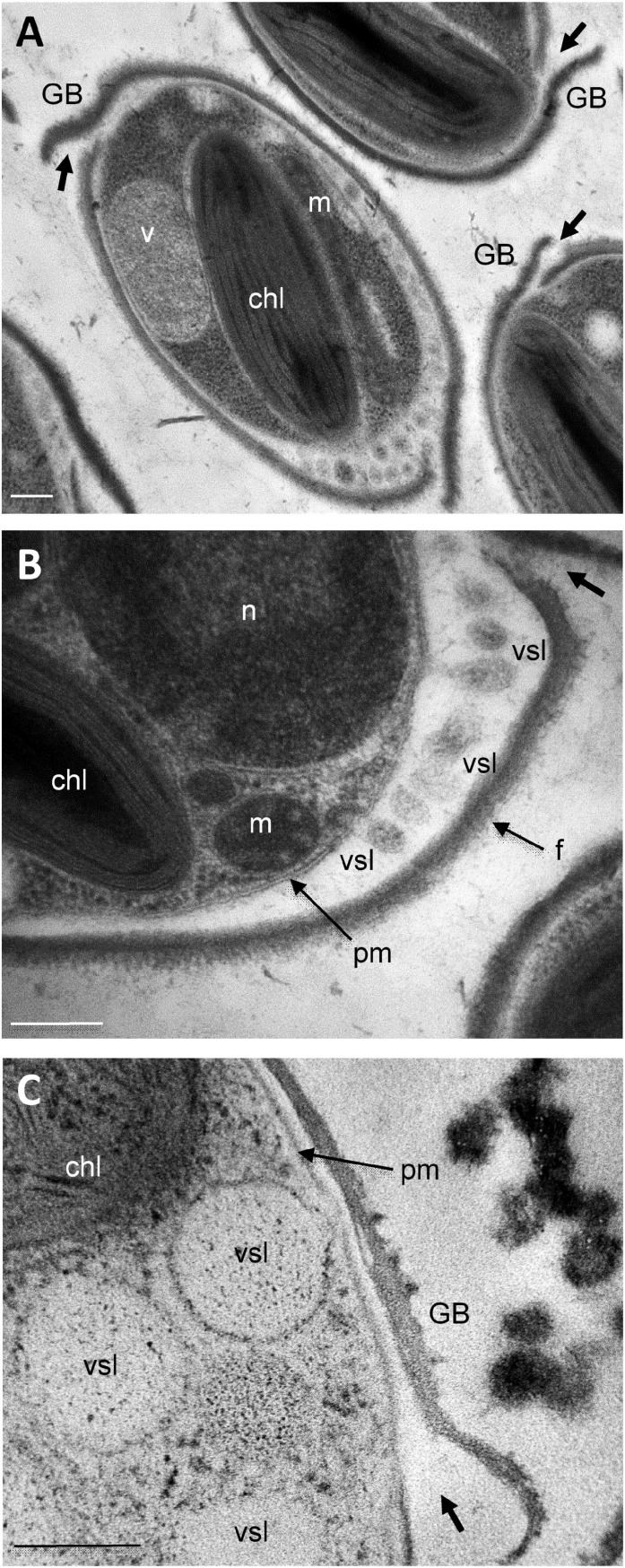
Ultrastructural characterization of the release site of secretory vesicles in *P. tricornutum* oval morphotype. Transmission Electron micrographs of Pt3 *Phaeodactylum tricornutum* oval morphotype illustrating the secretion through vesicles. **(A)** Whole view of the oval cell. **(B)** Magnification in the interspace between the plasma membrane and the frustule where secretory vesicles are accumulated. **(C)** Secretory vesicles nearby the girdle band. chl: plastid; GB: girdle band; m: mitochondria; n: nucleus; pm: plasma membrane; f: frustule; vsl: secretory vesicles v: vacuole. Bars, 0.2 μm.

As far as we know from the literature, the mechanisms of protein release in *P. tricornutum* cells has not been studied in details yet. Imaging oval cell at subcellular levels revealed frustule opening valves (thick arrow) that might be involved in the secretory process (Figure 8A). In particular, a number of electron-dense vesicles are accumulated in the interspace between the plasma membrane and the frustule (Figure 8B). Despite reduced cell volume, oval cells seem to be very active concerning secretion (Figure 8C). In the future, functional studies will be necessary to depict the complete transport mechanisms of proteins in the three morphotypes of *P. tricornutum* and the dynamics of protein release. This is of particular interest as *P. tricornutum* has been used recently to produce recombinant monoclonal antibodies. However, the production yield is insufficient to envision any industrial commercialization. Thus, gaining comprehension of *P. tricornutum* cellular and metabolism processes would be helpful in the future to maximize the use of *P. tricornutum* as a green alternative cell biofactory. The results presented in this study suggests that using oval cells for the production of biopharmaceutical proteins might be helpful to improve the production yield. In addition, characterizing the secretory pathways by which proteins such as recombinant mAbs are released would be of particular interest in this blue biotech context.

## Conclusion

The work described herein revealed characteristics of cellular organelles, cytoskeleton and protein secretion in the three main morphotypes of *P. tricornutum* Pt3 fusiform, oval and triradiate.

The three morphotypes share similarities including spectral characteristics of the plastid, the location of the nucleus, the organization of mitochondria around the plastid as well as the existence of both a F-actin cortex and an intracellular network of F-actin. In contrast, the oval cell, which is the smallest Pt3 morphotype, presents a thick frustule and a plastid occupying a large cell volume. As compare to fusiform and triradiate cells, oval cells release spontaneously proteins more rapidly. In addition, comparison of whole transcriptomes of oval versus fusiform or triradiate cells revealed numerous differential expression of positive and negative regulators belonging to the complex dynamic secretory machinery. Since such processes are mostly regulated at the protein level, future proteomic analyses is required to gain informations regarding the fine regulation of secretion in the three Pt3 morphotypes.

This study highlights the specificities occurring within the oval morphotype confirming that the oval cells secrete more rapidly proteins. Thus, in the future, using oval cells for the production of biopharmaceutical proteins might be helpful to improve the production yield. Indeed, due to easy transformation procedure, *P. tricornutum* fusiform cells are currently used to produce recombinant mAbs directed against viruses. However, the production yield of the secreted recombinant mAbs is still low (2 mg L^–1^) and insufficient to envision an industrial commercialization (Hempel and Maier, 2012). In addition, characterizing the secretory pathway(s) by which proteins such as recombinant mAbs are released would be of particular interest and will help maximizing the future use of *P. tricornutum* as a green alternative cell biofactory.

## Data Availability Statement

The original contributions presented in the study are included in the article/Supplementary Material, further inquiries can be directed to the corresponding author/s.

## Author Contributions

LG, MBa, and CB: concept and design of the research and writing of the manuscript. LG, CB, DS, SB, and MBé: experimental work. LG, MBa, CB, MR, DS, and MBé: data analysis and interpretation. All authors have read, corrected, and agreed on the content of the manuscript prior to its submission.

## Conflict of Interest

The authors declare that the research was conducted in the absence of any commercial or financial relationships that could be construed as a potential conflict of interest.

## Footnotes

1 https://www.uniprot.org

## References

[B1] Abràmoff M. D. Magalhães P. J. Ram S. J. (2004). Image processing with ImageJ. *Biophot. Intern.* 11 36–43.

[B2] Apt K. E. Grossman A. R. Kroth-Pancic P. G. (1996). Stable nuclear transformation of the diatom *Phaeodactylum tricornutum*. *Molec. Gen. Genet.* 252 572–579. 8914518 10.1007/BF02172403

[B3] Baïet B. Burel C. Saint-Jean B. Louvet R. Menu-Bouaouiche L. Kiefer-Meyer M.-C. (2011). N-glycans of *Phaeodactylum tricornutum* diatom and functional characterization of its N-acetylglucosaminyltransferase I enzyme. *J. Biol. Chem.* 286 6152–6164. 10.1074/jbc.m110.175711 21169367 PMC3057864

[B4] Bailleul B. Berne N. Murik O. Petroutsos D. Prihoda J. Tanaka A. (2015). Energetic coupling between plastids and mitochondria drives CO_2_ assimilation in diatoms. *Nature* 524 366–369. 10.1038/nature14599 26168400

[B5] Bartual A. Gálvez J. A. Ojeda F. (2008). Phenotypic response of the diatom *Phaeodactylum tricornutum* Bohlin to experimental changes in the inorganic carbon system. *Bot. Mar.* 51 350–359.

[B6] Bezanilla M. (2013). What can plants do for cell biology? *Mol. Biol. Cell.* 24 2491–2493. 10.1091/mbc.e12-10-0706 23943803 PMC3744949

[B7] Borowitzka M. A. Volcani B. E. (1978). Polymorphic diatom *Phaeodactylum tricornutum*: ultrastructure of its morphotypes. *J. Phycol.* 14 10–21. 10.1111/j.1529-8817.1978.tb00625.x

[B8] Bowler C. Allen A. E. Badger J. H. Grimwood J. Jabbari K. Kuo A. (2008). The *Phaeodactylum* genome reveals the evolutionary history of diatom genomes. *Nature* 456 239–244. 18923393 10.1038/nature07410

[B9] Bowler C. Vardi A. Allen A. E. (2010). Oceanographic and biogeochemical insights from diatom genomes. *Annu. Rev. Mar. Sci.* 2 333–365. 10.1146/annurev-marine-120308-081051 21141668

[B10] Buhmann M. T. Schulze B. Förderer A. Schleheck D. Kroth P. G. (2016). Bacteria may induce the secretion of mucin-like proteins by the diatom *Phaeodactylum tricornutum*. *J. Phycol.* 52 463–474. 10.1111/jpy.12409 26993172

[B11] Butler T. Kapoore R. V. Vaidyanathan S. (2020). *Phaedactylum tricornutum*: a diatom cell factory. *Trends Biotechnol.* 38 606–622. 10.1016/j.tibtech.2019.12.023 31980300

[B12] Cadoret J.-P. Garnier M. Saint-Jean B. (2012). Microalgae, functional genomics and biotechnology. *Adv. Bot. Res.* 64 285–341. 10.1007/978-3-540-74335-4_17

[B13] Daboussi F. Leduc S. Maréchal A. Dubois G. Guyot V. Perez-Michaut C. (2014). Genome engineering empowers the diatom *Phaeodactylum tricornutum* for biotechnology. *Nat. Commun.* 5:3831. 10.1038/ncomms4831 24871200

[B14] D’Adamo S. Schiano di Visconte G. Lowe G. Szaub-Newton J. Beacham T. Landels A. (2019). Engineering the unicellular alga *Phaeodactylum tricornutum* for high-value plant triterpenoid production. *Plant Biotechnol. J.* 17 75–87. 10.1111/pbi.12948 29754445 PMC6330534

[B15] De Martino A. Bartual A. Willis A. Meichenin A. Villazán B. Maheswari U. (2011). Physiological and molecular evidence that environmental changes elicit morphological interconversion in the model diatom *Phaeodactylum tricornutum*. *Protist* 162 462–481. 10.1016/j.protis.2011.02.002 21600845

[B16] De Martino A. Meichenin A. Shi J. Pan K. Bowler C. (2007). Genetic and phenotypic characterization of *Phaeodactylum tricornutum* (*Bacillariophyceae*). accessions. *J. Phycol.* 43 992–1009. 10.1111/j.1529-8817.2007.00384.x

[B17] De Riso V. Raniello R. Maumus F. Rogato A. Bowler C. Falciatore A. (2009). Gene silencing in the marine diatom *Phaeodactylum tricornutum*. *Nucleic Acids Res.* 37:e96. 10.1093/nar/gkp448 19487243 PMC2724275

[B18] Depauw F. Rogato A. D’Alcalá M. Falciatore A. (2012). Exploring the molecular basis of responses to light in marine diatoms. *J. Exper. Bot.* 63 1575–1591. 10.1093/jxb/ers005 22328904

[B19] Dumontier R. Mareck A. Mati-Baouche N. Lerouge P. Bardor M. (2018). “Toward future engineering of the N-glycosylation pathways in microalgae for optimizing the production of biopharmaceuticals,” in *Microalgal Biotechnology*, eds Jacob-Lopes E. Zepka L. Q. Queiroz M. I. (London: IntechOpen), 10.5772/intechopen.73401

[B20] Erdene-Ochir E. Shin B. K. Kwon B. Jung C. Pan C. H. (2019). Identification and characterisation of the novel endogenous promoter HASP1 and its signal peptide from *Phaeodactylum tricornutum*. *Sci Rep.* 9:9941. 10.1038/s41598-019-45786-9 31289300 PMC6617621

[B21] Flori S. Jouneau P. H. Bailleul B. Gallet B. Estrozi L. F. Moriscot C. (2017). Plastid thylakoid architecture optimizes photosynthesis in diatoms. *Nat. Commun.* 8:15885. 10.1038/ncomms15885 28631733 PMC5481826

[B22] Francius G. Tesson B. Dague E. Martin-Jézéquel V. Dufrêne Y. F. (2008). Nanostructure and nanomechanics of live *Phaeodactylum tricornutum* morphotypes. *Environ. Microbiol.* 10 1344–1356. 10.1111/j.1462-2920.2007.01551.x 18248452

[B23] Galas L. Gallavardin T. Bénard M. Lehner A. Schapman D. Lebon A. (2018). ”Probe, Sample, and Instrument (PSI)”: the hat-trick for fluorescence live cell imaging. *Chemosensors* 6:40. 10.3390/chemosensors6030040

[B24] Gómez-Loredo A. Benavides J. Rito-Palomares M. (2016). Growth kinetics and fucoxanthin production of *Phaeodactylum tricornutum* and *Isochrysis galbana* cultures at different light and agitation conditions. *J. Appl. Phycol.* 28 849–860. 10.1007/s10811-015-0635-0

[B25] Gutenbrunner S. A. Thalhamer J. Schmid A.-M. M. (1994). Proteinaceous and immunochemical distinctions between the oval and fusiform morphotypes of *Phaeodactylum tricornutum* (*Bacillariophyceae*). *J. Phycol.* 30 129–136. 10.1111/j.0022-3646.1994.00129.x

[B26] Hamilton M. L. Warwick J. Terry A. Allen M. J. Napier J. A. Sayanova O. (2015). Towards the industrial production of omega-3 long chain polyunsaturated fatty acids from a genetically modified diatom *Phaeodactylum tricornutum*. *PLoS One* 10:e0144054. 10.1371/journal.pone.0144054 26658738 PMC4681182

[B27] He L. Han X. Yu Z. (2014). A Rare *Phaeodactylum tricornutum* cruciform morphotype: culture conditions, transformation and unique fatty acid characteristics. *PLoS One* 9:e93922. 10.1371/journal.pone.0093922 24710200 PMC3977982

[B28] Hempel F. Lau J. Klingl A. Maier U. G. (2011). Algae as protein factories: expression of a human antibody and the respective antigen in the diatom *Phaeodactylum tricornutum*. *PLoS One* 6:e28424. 10.1371/journal.pone.0028424 22164289 PMC3229587

[B29] Hempel F. Maier U. G. (2012). An engineered diatom acting like a plasma cell secreting human IgG antibodies with high efficiency. *Microb. Cell. Fact.* 11:126. 10.1186/1475-2859-11-126 22970838 PMC3503769

[B30] Hempel F. Maier U. G. (2016). Microalgae as solar-powered protein factories. *Adv. Exp. Med. Biol.* 896 241–262. 10.1007/978-3-319-27216-0_16 27165330

[B31] Hempel F. Maurer M. Brockmann B. Mayer C. Biedenkopf N. Kelterbaum A. (2017). From hybridomas to a robust microalgal-based production platform: molecular design of a diatom secreting monoclonal antibodies directed against the Marburg virus nucleoprotein. *Microb. Cell. Fact.* 16:131. 10.1186/s12934-017-0745-2 28750668 PMC5531009

[B32] Herbstová M. Bína D. Kaòa R. Vácha F. Litvín R. (2017). Red-light phenotype in a marine diatom involves a specialized oligomeric red-shifted antenna and altered cell morphology. *Sci. Rep.* 7:11976. 10.1038/s41598-017-12247-0 28931902 PMC5607283

[B33] Karas B. J. Diner R. E. Lefebvre S. C. McQuaid J. Phillips A. P. R. Noddings C. M. (2015). Designer diatom episomes delivered by bacterial conjugation. *Nat. Commun.* 6:6925. 10.1038/ncomms7925 25897682 PMC4411287

[B34] Keeling P. J. Palmer J. D. (2008). Horizontal gene transfer in eukaryotic evolution. *Nat. Rev. Genet.* 9 605–618. 10.1038/nrg2386 18591983

[B35] Kuczynska P. Jemiola-Rzeminska M. Strzalka K. (2015). Photosynthetic pigments in diatoms. *Mar. Drugs* 13 5847–5881. 10.3390/md13095847 26389924 PMC4584358

[B36] Lamote M. Darko E. Schoefs B. Lemoine Y. (2003). Assembly of the photosynthetic apparatus in embryos from *Fucus serratus* L. *Photosynth. Res.* 77 45–52. 10.1023/A:1024999024157 16228383

[B37] León-Bañares R. González-Ballester D. Galván A. Fernández E. (2004). Transgenic microalgae as green cell-factories. *Trends Biotechnol.* 22 45–52. 10.1016/j.tibtech.2003.11.003 14690622

[B38] Levitan O. Chen M. Kuang X. Cheong K. Y. Jiang J. Banal M. (2019). Structural and functional analyses of photosystem II in the marine diatom *Phaeodactylum tricornutum*. *Proc. Natl. Acad. Sci. U.S.A.* 116 17316–17322. 10.1073/pnas.1906726116 31409711 PMC6717305

[B39] Lewin J. C. Lewin R. A. Philpott D. E. (1958). Observations on *Phaeodactylum tricornutum*. *J. Gen. Microbiol.* 18 418–426. 13525658 10.1099/00221287-18-2-418

[B40] Liu X. J. Hempel F. Stork S. Bolte K. Moog D. Heimerl T. (2016). Addressing various compartments of the diatom model organism *Phaeodactylum tricornutum* via sub-cellular marker proteins. *Algal Res.* 20 249–257. 10.1016/j.algal.2016.10.018

[B41] Lupette J. Jaussaud A. Seddiki K. Morabito C. Brugière S. Schaller H. (2019). The architecture of lipid droplets in the diatom *Phaeodactylum tricornutum*. *Algal Res.* 38:101415. 10.1016/j.algal.2019.101415

[B42] Mann M. Serif M. Jakob T. Kroth P. G. Wilhelm C. (2017). PtAUREO1a and PtAUREO1b knockout mutants of the diatom *Phaeodactylum tricornutum* are blocked in photoacclimation to blue light. *J. Plant Physiol.* 217 44–48. 10.1016/j.jplph.2017.05.020 28610707

[B43] Martin S. G. (2014). Yeasts as models in cell biology. *FEMS Microbiol. Rev.* 38:143. 10.1111/1574-6976.12068 24606190

[B44] Martin-Jézéquel V. Tesson B. (2013). “*Phaeodactylum tricornutum* polymorphism: an overview,” in *Advances in Algal Cell Biology*, eds Heimann K. Katsaros C. (Berlin: De Gruyter), 43–80.

[B45] Mathieu-Rivet E. Kiefer-Meyer M. C. Vanier G. Ovide C. Burel C. Lerouge P. (2014). Protein N-glycosylation in eukaryotic microalgae and its impact on the production of nuclear expressed biopharmaceuticals. *Front. Plant Sci.* 5:359. 10.3389/fpls.2014.00359 25183966 PMC4135232

[B46] Mathur M. Xiang J. S. Smolke C. D. (2017). Mammalian synthetic biology for studying the cell. *J. Cell. Biol.* 216 73–82. 10.1083/jcb.201611002 27932576 PMC5223614

[B47] McClure D. D. Luiz A. Gerber B. Barton G. W. Kavanagh J. M. (2018). An investigation into the effect of culture conditions on fucoxanthin production using the marine microalgae *Phaeodactylum tricornutum*. *Algal Res.* 29 41–48. 10.1016/j.algal.2017.11.015

[B48] Miyahara M. Aoi M. Inoue-Kashino N. Kashino Y. Ifuku K. (2013). Highly efficient transformation of the diatom *Phaeodactylum tricornutum* by multi-pulse electroporation. *Biosci. Biotechnol. Biochem.* 77 874–876. 10.1271/bbb.120936 23563551

[B49] Moustafa A. Beszteri B. Maier U. G. Bowler C. Valentin K. Bhattacharya D. (2009). Genomic footprints of a cryptic plastid endosymbiosis in diatoms. *Science* 324 1724–1726. 10.1126/science.1172983 19556510

[B50] Niu Y. F. Yang Z. K. Zhang M. H. Zhu C. C. Yang W. D. Liu J. S. (2012). Transformation of diatom *Phaeodactylum tricornutum* by electroporation and establishment of inducible selection marker. *Biotechniques* 52 1–3. 10.2144/000113881 26307256

[B51] Nymark M. Sharma A. Sparstad T. Bones A. M. Winge P. (2016). A CRISPR/Cas9 system adapted for gene editing in marine algae. *Sci. Rep.* 6:24951. 10.1038/srep24951 PMC4842962 27108533

[B52] Nymark M. Valle K. C. Hancke K. Winge P. Andresen K. Johnsen G. (2013). Molecular and photosynthetic responses to prolonged darkness and subsequent acclimation to Re-illumination in the diatom *Phaeodactylum tricornutum*. *PLoS One* 8:e58722. 10.1371/journal.pone.0058722 23520530 PMC3592843

[B53] Ovide C. Kiefer-Meyer M. C. Bérard C. Vergne N. Lecroq T. Plasson C. (2018). Comparative in depth RNA sequencing of *P. tricornutum*’s morphotypes reveals specific features of the oval morphotype. *Sci. Rep.* 8:14340. 10.1038/s41598-018-32519-7 30254372 PMC6156597

[B54] Premvardhan L. Réfrégiers M. Büchel C. (2013). Pigment organization effects on energy transfer and Chl a emission imaged in the diatoms *C. meneghiniana* and *P. tricornutum in vivo*: a confocal laser scanning fluorescence (CLSF). microscopy and spectroscopy study. *J. Phys. Chem.* 117 11272–11281. 10.1021/jp402094c 23844975

[B55] Prihoda J. Tanaka A. de Paula W. B. M. Allen J. F. Tirichine L. Bowler C. (2012). Chloroplast-mitochondria cross-talk in diatoms. *J. Exp. Bot.* 63 1543–1557. 22268145 10.1093/jxb/err441

[B56] Rasband W. S. (1997–2018). *ImageJ.* Bethesda, MA: U.S. National Institutes of Health. Available online at: https://imagej.nih.gov/ij/

[B57] Rastogi A. Maheswari U. Dorrell R. G. Vieira F. R. J. Maumus F. Kustka A. (2018). Integrative analysis of large scale transcriptome data draws a comprehensive landscape of *Phaeodactylum tricornutum* genome and evolutionary origin of diatoms. *Sci. Rep.* 8:4834. 10.1038/s41598-018-23106-x 29556065 PMC5859163

[B58] Rosales-Mendoza S. Solís-Andrade K. I. Márquez-Escobar V. A. González-Ortega O. Bañuelos-Hernandez B. (2020). Current advances in the algae-made biopharmaceuticals field. *Expert Opin. Biol.* 20 751–766. 10.1080/14712598.2020.1739643 32142617

[B59] Sapriel G. Quinet M. Heijde M. Jourdren L. Tanty V. Luo G. (2009). Genome-wide transcriptome analyses of silicon metabolism in *Phaeodactylum tricornutum* reveal the multilevel regulation of silicic acid transporters. *PLoS One* 4:e7458. 10.1371/journal.pone.0007458 19829693 PMC2758714

[B60] Sasso S. Pohnert G. Lohr M. Mittag M. Hertweck C. (2012). Microalgae in the postgenomic era: a blooming reservoir for new natural products. *FEMS Microbiol. Rev.* 36 761–785. 10.1111/j.1574-6976.2011.00304.x 22091538

[B61] Scala S. Carels N. Falciatore A. Chiusane M. L. Bowler C. (2002). Genome properties of the diatom *P. tricornutum*. *Plant Physiol.* 129 996–1002. 10.1104/pp.010713 PMC166495 12114555

[B62] Serif M. Dubois G. Finoux A. L. Teste M. A. Jallet D. Daboussi F. (2018). One-step generation of multiple gene knock-outs in the diatom *Phaeodactylum tricornutum* by DNA-free genome editing. *Nat. Commun.* 9:3924. 10.1038/s41467-018-06378-9 PMC6156588 30254261

[B63] Serif M. Lepetit B. Weissert K. Kroth P. G. Rio Bartulos C. (2017). A fast and reliable strategy to generate TALEN-mediated gene knockouts in the diatom *Phaeodactylum tricornutum*. *Algal Res.* 23 186–195. 10.1016/j.algal.2017.02.005

[B64] Siaut M. Heijde M. Mangogna M. Montsant A. Coesel S. Allen A. (2007). Molecular toolbox for studying diatom biology in *Phaeodactylum tricornutum*. *Gene* 406 23–35. 10.1016/j.gene.2007.05.022 17658702

[B65] Slattery S. S. Diamond A. Wang H. Therrien J. A. Lant J. T. Jazey T. (2018). An expanded plasmid-based genetic toolbox enables cas9 genome editing and stable maintenance of synthetic pathways in *Phaeodactylum tricornutum*. *ACS Synth. Biol.* 7 328–338. 10.1021/acssynbio.7b00191 29298053

[B66] Song Z. Lye G. J. Parker B. M. (2020). Morphological and biochemical changes in *Phaedactylum tricornutum* triggered by culture media: Implications for industrial exploitation. *Algal Res.* 47:101822. 10.1016/j.algal.2020.101822

[B67] Stukenberg D. Zauner S. Dell’Aquila G. Maier U. G. (2018). Optimizing CRISPR/Cas9 for the diatom *Phaeodactylum tricornutum*. *Front. Plant Sci.* 9:740. 10.3389/fpls.2018.00740 29928285 PMC5998643

[B68] Tanaka A. De Martino A. Amato A. Montsant A. Mathieu B. Rostaing P. (2015). Ultrastructure and membrane traffic during cell division in the marine pennate diatom *Phaeodactylum tricornutum*. *Protist* 166 506–521. 10.1016/j.protis.2015.07.005 26386358 PMC4710849

[B69] Tesson B. Hildebrand M. (2010). Extensive and intimate association of the cytoskeleton with forming silica in diatoms: control over patterning on the meso- and micro-scale. *PLoS One* 10:e14300. 10.1371/journal.pone.0014300 21200414 PMC3000822

[B70] Uwizeye C. Decelle J. Jouneau P.-H. Gallet B. Keck J.-B. Moriscot C. (2020). In-cell quantitative structural imaging of phytoplankton using 3D electron microscopy. *bioRxiv* [Preprint], 10.1101/2020.05.19.104166

[B71] Vanier G. Hempel F. Chan P. Rodamer M. Vaudry D. Maier U. G. (2015). Biochemical characterization of human anti-Hepatitis B monoclonal antibody produced in the microalgae *Phaeodactylum tricornutum*. *PLoS One* 10:e0139282. 10.1371/journal.pone.0139282 26437211 PMC4593558

[B72] Vanier G. Stelter S. Vanier J. Hempel F. Maier U. G. Lerouge P. (2018). Alga-made anti-Hepatitis B antibody binds to human Fcγ receptors. *Biotechnol. J.* 13:e1700496. 10.1002/biot.201700496 29194986

[B73] Vartanian M. Desclés J. Quinet M. Douady S. Lopez P. J. (2009). Plasticity and robustness of pattern formation in the model diatom *Phaeodactylum tricornutum*. *New Phytol.* 182 429–442. 10.1111/j.1469-8137.2009.02769.x 19210721

[B74] Veith T. Bühel C. (2007). The monomeric photosystem I-complex of the diatom *Phaeodactylum tricornutum* binds specific fucoxanthin chlorophyll proteins (FCPs) as light-harvesting complexes. *Biochim. Biophys. Acta* 1767 1428–1435. 10.1016/j.bbabio.2007.09.004 18028870

[B75] Willis A. Chiovitti A. Dugdale T. M. Wetherbee R. (2013). Characterization of the extracellular matrix of *Phaeodactylum tricornutum* (*bacillariophyceae*): Structure, composition, and adhesive characteristics. *J. Phycol.* 49 937–949. 10.1111/jpy.12103 27007317

[B76] Wilson D. P. (1946). The triradiate and other forms of *Nitschia closterum* (Ehrenberg). Wm. Smith form *Minutissima* of Allen and Nelson. *J. Mar. Biol. Assoc.* 26 235–270. 10.1017/s002531540001211x

[B77] Wong D. N. Franz A. K. (2013). A comparison of lipid storage in *Phaeodactylum tricornutum* and *Tetraselmis suecica* using laser scanning confocal microscopy. *J. Microbiol. Methods* 95 122–128. 10.1016/j.mimet.2013.07.026 23933493

[B78] Zaslavskaia L. A. Lippmeier J. C. Kroth P. G. Grossman A. R. Apt K. E. (2000). Transformation of the diatom *Phaeodactylum tricornutum* (*Bacillariophyceae*). with a variety of selectable marker and reporter genes. *J. Phycol.* 36 379–386. 10.1046/j.1529-8817.2000.99164.x

[B79] Zhang C. Hu H. (2014). High-efficiency nuclear transformation of the diatom *Phaeodactylum tricornutum* by electroporation. *Mar. Genomics* 16 63–66. 10.1016/j.margen.2013.10.003 24269346

[B80] Zou L. Chen J. Zheng D. Balamurugan S. Li D. W. Yang W. D. (2018). High-efficiency promoter-driven coordinated regulation of multiple metabolic nodes elevates lipid accumulation in the model microalga *Phaeodactylum tricornutum*. *Microb. Cell. Fact.* 17:54. 10.1186/s12934-018-0906-y 29618383 PMC5885374

