# Supplementary material for: Comparative Structural and Functional Analyses of the Fusiform, Oval, and Triradiate Morphotypes of Phaeodactylum tricornutum Pt3 Strain
Source: Front Plant Sci. 2021 Apr 12;12:638181. doi: 10.3389/fpls.2021.638181 (PMC8072121; doi:10.3389/fpls.2021.638181)
Supplement: Supplementary file 3 [file Data_Sheet_1.DOCX]

Supplementary Material

# Supplementary Figures and Tables

## Supplementary Figures

**
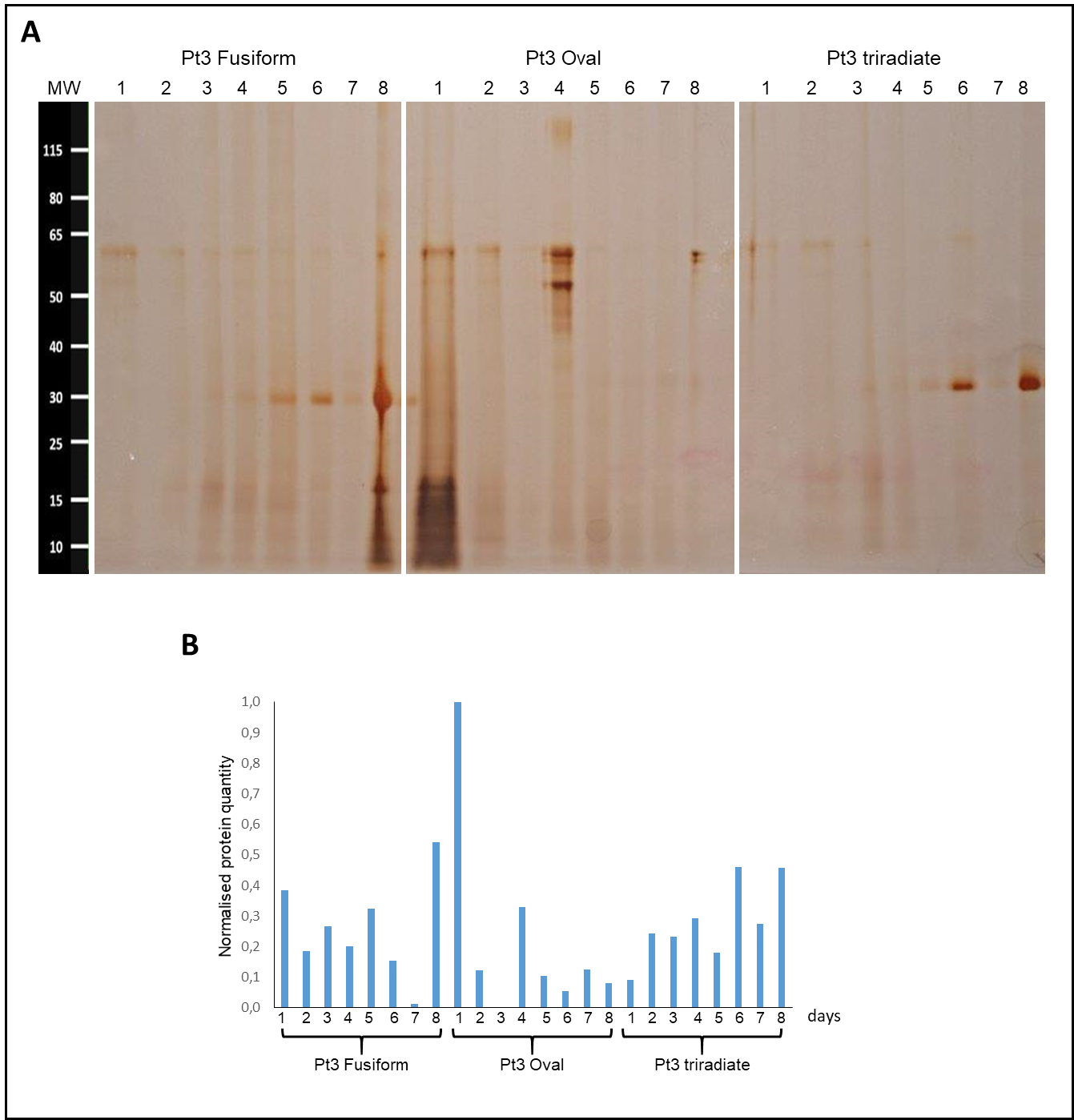
**

**Supplementary Figure 1: 8 day-kinetic of protein secretion for the three morphotypes of *P. tricornutum***

**A**: SDS-PAGE gel analysis of secreted proteins from Pt3 morphotypes over eight days of culture. Proteins from the culture medium were separated on a 4-12% Bis-Tris gel followed by silver staining.

Lane MW: Molecular Weight Ladder; lanes 1 to 8: corresponding day for culture medium harvesting.

**B**: Histograms presenting the normalized quantity of released proteins from the three Pt3 morphotypes over eight days of culture.

## Supplementary Tables

**Supplementary Tables have been submitted online as EXCEL files. This includes the following tables:**

**Supplementary Table 1:** Summary of the genes encoding for proteins involved in the kinesin complex or other proteins known to be associated or to move along the microtubules that are expressed in the oval cells compared to the fusiform and triradiate ones. The information have been retrieved from the full dataset reported in Ovide et al., 2018.

**Supplementary Table 2:** List of the genes encoded for molecular actors involved in the vesicular transport along the secretory pathway that are expressed in the oval cells compared to the fusiform and triradiate ones. The information have been retrieved from the full dataset reported in Ovide et al., 2018.

**
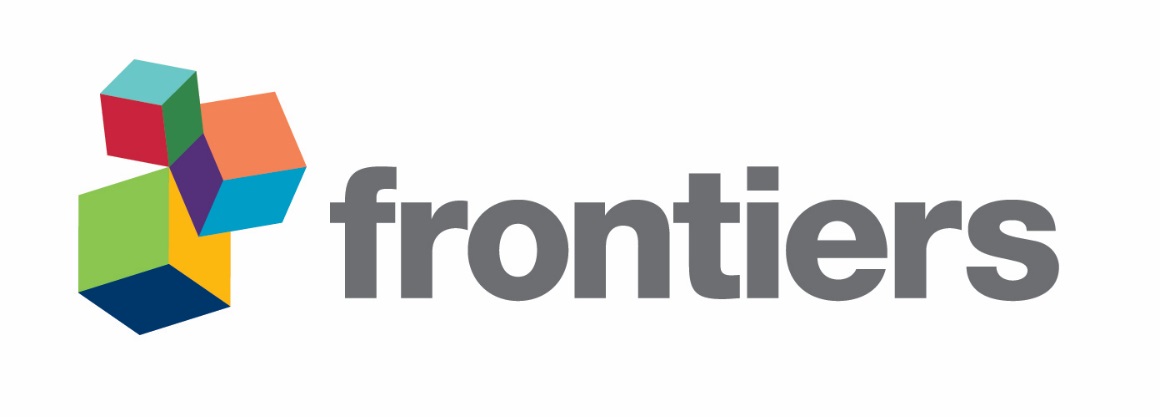
**
